# Examining Stereotype Threat in Neuropsychological Testing: A Usability and User Experience Pilot Study

**DOI:** 10.1093/geroni/igab046.2474

**Published:** 2021-12-17

**Authors:** Jazlyn Armendariz, Sarah Hwang, Giovanna Garrido Blanco, Maria Pena, Thomas Chan

**Affiliations:** 1 California State University Northridge, Northridge, California, United States; 2 California State University Northridge, Chanhassen, Minnesota, United States; 3 California State University Northridge, Pacoima, California, United States

## Abstract

Stereotype threat is defined as the situational predicament when people feel at risk of conforming to social stereotypes. Correspondingly, stereotype threat may negatively impair a persons’ working memory and cognitive abilities during neuropsychological tests due to hyper awareness of negative stereotypes. Moreover, it is critical to test the usability and the user experience of application-based neuropsychological assessments within diverse aging adult populations. In this pilot study, verbal expressions of feeling pressure to succeed, within a diverse population of young adults, were examined while taking an application-based neuropsychological assessment. Data was collected from 15 self-identified respondents (i.e., 7 Latinx, 5 Asian, 3 Bi-racial). Before beginning the assessment, 8 out of 15 participants exhibited self-handicapping behaviors such as offering explanations of mental exhaustion due to work and lack of sleep. Literature suggests these expressions are related to the onset of anxiety prior to taking cognitive tests, and contribute to potentially offering an excuse in anticipation of poor performance. Additionally, 3 out of 15 participants noted that even though the tasks were simple, they felt unintelligent because they did not complete the tasks to their best abilities (e.g., “I felt stupid. It was simple”). Findings from this pilot support the negative impact stereotype threats have on feelings of inadequacy and increase of anxiety levels among ethnic minorities in testing settings. Further emphases on examining the usability and user experience of application-based tests are needed, particularly within a diverse population of aging adults to facilitate more culturally competent neuropsychological testing experiences.

